# Supplementary material for: Environmental inefficiencies for arrival flights at European airports
Source: PLoS One. 2023 Jun 23;18(6):e0287612. doi: 10.1371/journal.pone.0287612 (PMC10289470; doi:10.1371/journal.pone.0287612)
Supplement: Attachment — Submitted filename: response_reviewer_comments.pdf [file pone.0287612.s001.pdf]

Editorial comments:

1. Please ensure that your manuscript meets PLOS ONE's style requirements, including those for file naming.

Response: We followed the style guidelines defined in the latex template.

2. We note that Figure 3 and 4 in your submission contain [map/satellite] images which may be copyrighted.

Response: The figures are generated by the authors specifically for this paper. There is no copyright issues.

3. Please review your reference list to ensure that it is complete and correct.

Response: We double-checked the reference list.

Reviewer #1:

Accurate article from a pilot perspective with correct aviation terminology. It indicates how different descent patterns influence emissions and fuel consumptions. However some airports like EGLL presents constraints - I.e. London TMA airspace - which make the use of CDO not practical, preventing the optimisation of emissions and fuel consumption.

An useful recommendation to include, which may go beyond the scope of the article but provide some practical implications, could indicate how airports and ATC can improve the design of descent patterns taking into account the various constraints, peculiar of each airport and airspace.

Response: We thank the reviewer for the feedback. In the revised paper, we added in the discussion section (Section 6) our recommendations on how airports and ATC can improve the design for arriving flights.

Reviewer #2:

This manuscript is well written and easy to understand. Each section has the appropriate amount of detail, and the figures & tables are well made.

Response: We thank the reviewer for the feedback.

I have the following comments to help further improve the manuscript.

1. Page 7, line 3 "merge point with a DIRECT when spacing is obtained." – Please clarify what DIRECT is in this case

Response: We clarified the sentence in the manuscript.

2. Page 8, last paragraph, line 3 "area of interest, 50nm by default, 60nm for EGLC, 90nm for LFPG". How are these sizes determined?

Response: These sizes are determined based on the radius, beyond which the capacity management procedures occur (e.g.:. point-merges and holdings). These are different from each airport and have been selected based on exploration of the flight data and airport arrival procedures. (This explanation is also included in the updated paper)

3. Page 9, Fig 6 – The boxplots show quite a few outliers, especially at EGLL airport. These outliers seem to have very large values for duration, normalized distance, fuel consumption, and emissions. What are the reasons for these outliers?

Response: The is mostly caused by the capacity of the airport. Depending on how busy of the airport, aircraft can stay in holding stack for many durations.

4. Page 9, Section 4.2, paragraph 2 "The calculation of fuel and emissions are all based on the mass that is 90 % of the maximum landing weight for the specific aircraft type." – Is this a reasonable assumption? Please add some more details or a reference to justify this assumption.

Response: This is indeed an assumption that we made to enable the same baseline for emission calculations for different airports. In the absence of real aircraft weight, such simplification is proposed to reduce the uncertainty across different airlines. We agree further research would incorporate more accurate estimation of mass at top of descent to increase the accuracy of the analysis. (This recommendation is also added to the update paper)

5. Page 10, Fig 7 – There seem to be 4 (1 point merge, 3 non-point merge) outliers that deviate from the general trend. These seem to have very low normalized fuel consumption even though their distance is high. Please add a discussion of these outliers in the manuscript text

Thank you for spotting the anomaly, we missed it in our study! We investigated those, and they are trajectories with anomalies in the lat/lon positions (see below) that were undetected during the preprocessing. We chose the option to be honest about it and

added few lines in the manuscript to explain. (The figure below is just a screenshot from our library, it will not be included in the manuscript as it could be just confusing to the reader without extensive explanation)

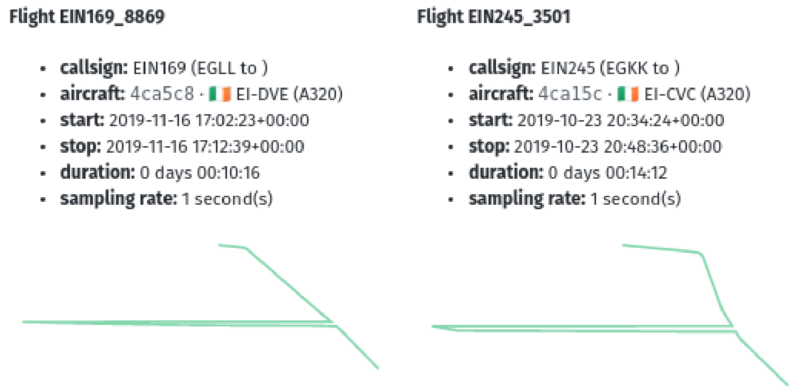

6. Page 10, 2nd paragraph below Fig 7 "p-value $\ll$ 0.0" – Please check if there a typo here. For t-tests, the p value is always positive so it cannot be $\ll$ 0.0.

Response: Thank you for pointing this out. It is indeed a typo. It should be "p-value $\ll$ 0.01", which is fixed now in the paper.
